# A porcine spectral assay library to quantify brain proteome by DIA-MS

**DOI:** 10.1038/s41597-026-07785-0

**Published:** 2026-08-24

**Authors:** Jakub Cervenka, Rita Sucha, Jirina Tyleckova, Ievgeniia Poliakh, Jaromir Novak, Eva Kotrcova, Karel Harant, Pavel Rehulka, Petr Vodicka

**Affiliations:** 1 Laboratory of Applied Proteome Analyses, Research Center PIGMOD, Institute of Animal Physiology and Genetics of the Czech Academy of Sciences, Libechov, Czechia; 2 Laboratory of Proteomics, Institute of Biochemistry and Experimental Oncology, First Faculty of Medicine, Charles University, Prague, Czechia; 3 Mass Spectrometry of Biopolymers, Institute of Organic Chemistry and Biochemistry of the Czech Academy of Sciences, Prague, Czechia; 4 Department of Molecular Pathology and Biology, Military Faculty of Medicine, University of Defence, Hradec Kralove, Czechia

## Abstract

Neurological disorders are the leading cause of health loss worldwide. The growing number of patients suffering from such conditions calls for improved strategies for their prevention, diagnosis, and therapy. To better understand human pathologies, relevant models and methodologies must be made available. In this study, we focused on a biomedical model capable of recapitulating the complexity of human pathology, the pig (*Sus scrofa*). Brain tissue and cerebrospinal fluid samples from a transgenic minipig model of Huntington’s disease were subjected to multiple extraction and fractionation steps. A proteomic mass spectrometry (MS) methodology then allowed the generation of a porcine spectral library for 8,321 proteins. Using data-independent acquisition (DIA), we demonstrated that our porcine spectral library substantially enhanced the quantitative potential of this untargeted MS approach, generating reproducible proteome-wide data. The porcine library also provides a comprehensive resource for the development of targeted MS assays, enabling the quantification of selected proteins with a key role not only in neuroscience.

## Background & Summary

The central nervous system (CNS) is susceptible to a wide range of disorders, including trauma, stroke, infection, cancer, and degeneration. These neurological conditions represent a leading cause of disease burden worldwide, with an impact on approximately one-third of the global population. The rising prevalence of neurological disorders highlights the urgent need for improved strategies in their prevention, diagnosis, and treatment^1^. Substantial efforts have been dedicated to advancing our understanding of underlying mechanisms and identifying novel diagnostic and prognostic biomarkers for neurological diseases. Proteomics offers valuable insights into the dynamics of physiological and pathological processes at the phenotypic level. In this context, large-scale proteomic analyses have been conducted on human post-mortem brain tissue samples^2–4^. To investigate early, pre-symptomatic disease stages, rodent animal models of various neurodegenerative diseases have been established^5^. Rodent models are widely used in drug screening, but novel approaches for disease prevention or therapy monitoring require animal models that more accurately recapitulate the complexity of human anatomy and disease pathology.

The pig (*Sus scrofa*) was introduced as a biomedical model organism for neuroscience several decades ago^6^. As a large animal model, the pig shares key anatomical, physiological, and immunological similarities with humans. Compared to non-human primates, the pig offers practical advantages in breeding and handling^7^. Over the past decades, various genetically modified porcine models have been developed to study neurological disorders, e.g., Alzheimer’s disease, amyotrophic lateral sclerosis, Huntington’s disease (HD), Parkinson’s disease, and traumatic brain injury (for review, see^8–11^). A transgenic (TG) minipig model of HD has been developed at our institute^12^ and utilised in preclinical gene therapy studies. These studies demonstrated that the administration of AAV5-miHTT effectively lowered the mutated huntingtin (mHTT) expression in minipig brain regions^13,14^, indicating the translational relevance of this model and the potential of this therapeutic approach, which is currently in Phase I/II clinical trials with promising outcomes in HD patients (https://uniqure.gcs-web.com/news-releases/news-release-details/uniqure-announces-positive-topline-results-pivotal-phase-iii).

Despite the porcine genome sequence being available since 2012^15^, this animal model lacks a sufficiently curated reference protein database. The Swiss-Prot database covers almost 99% of the human proteome and 79% of the mouse proteome based on gene count, but the porcine proteome coverage remains at only 6% (1,459 proteins)^16^. This limitation hampers the interpretation of proteome data based on mass spectrometry (MS), but can be overcome using the UniProtKB reference proteome. In addition to expertly reviewed proteins from Swiss-Prot, UniProtKB reference proteomes include entries from the automatically annotated TrEMBL database. By limiting the number of protein sequences to one per gene, the error rate for reference databases, which naturally contain frequent duplications, missing sequences, or misannotations, can be significantly reduced. Such a streamlined porcine UniProtKB reference proteome with protein entries mapping accurately to species-specific genes then enables more robust, system-wide proteomic analyses.

In bottom-up proteomics, proteins are enzymatically digested into peptides, which are then separated by liquid chromatography and analysed via tandem mass spectrometer (LC-MS/MS). Over the past two decades, various data-independent acquisition (DIA) methodologies have been developed, establishing DIA-MS as the method of choice for untargeted proteomic studies^17^. DIA-MS provides broad proteome coverage, quantitative accuracy, and reproducibility^18^. DIA data analysis generally follows one of the two main strategies: library-based or library-free^19^. The library-based approach utilises spectral libraries generated either *in silico* or from previous data-dependent acquisition (DDA) measurements. This approach has demonstrated higher quantitative reproducibility than the library-free approach^20^, but standing alone may result in lower proteome coverage^21^.

**Fig. 1:**
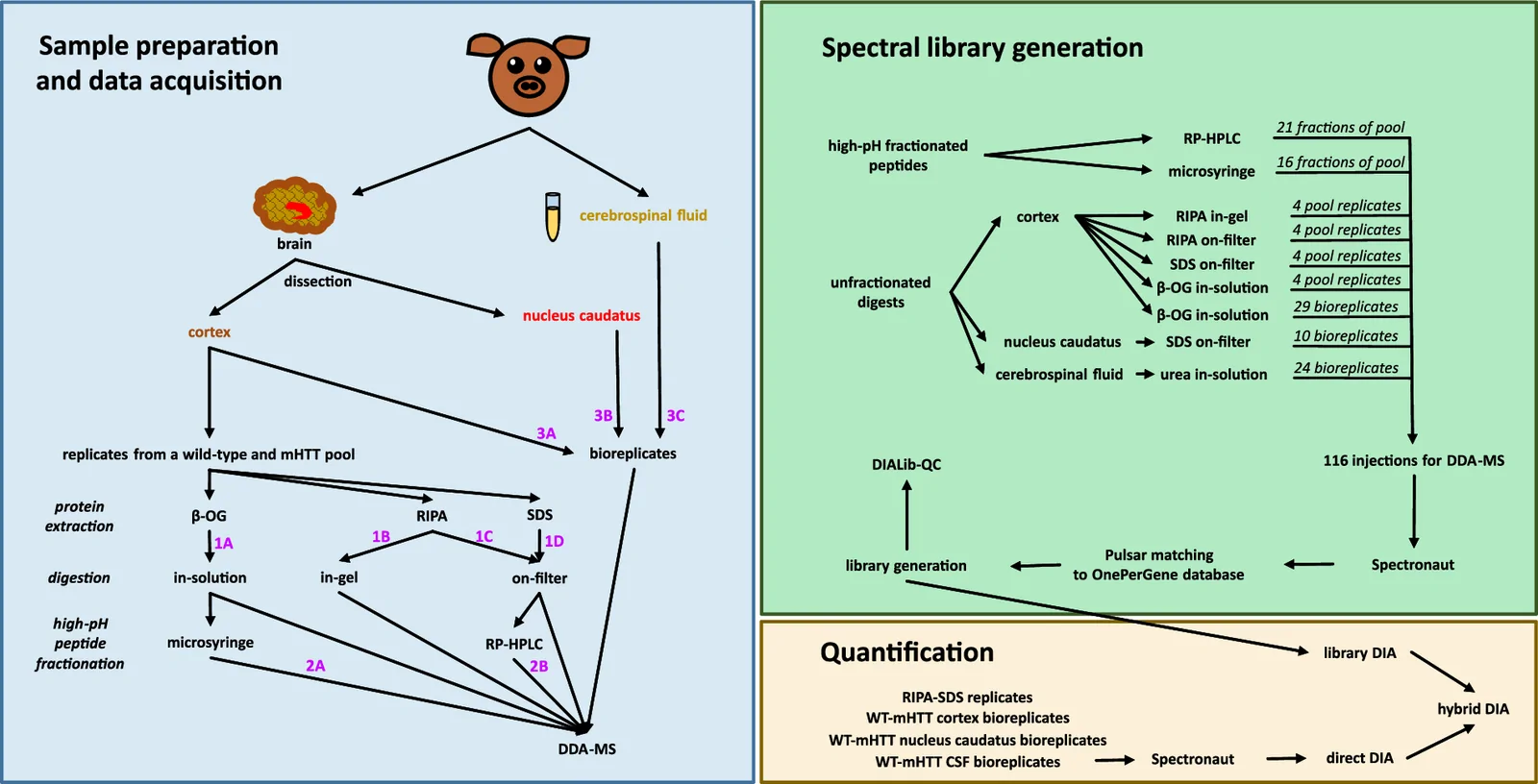
The experimental workflow used to generate and validate the porcine spectral library. The number of samples subjected to the different extraction and fractionation steps, including their tissue type, animal age, and *HTT* genotype is provided in Table 1. Violet labels indicate the protocol used and described in the Methods. β-OG, octyl β-D-glucopyranoside; CSF, cerebrospinal fluid; mHTT, mutated *HTT*; RIPA, radioimmunoprecipitation assay buffer; RP-HPLC, reversed-phase high-performance liquid chromatography; SDS, sodium dodecyl sulfate; WT, wild-type.

In this study, we present a spectral library with deep proteome coverage enabling routine analyses of the porcine brain proteome by DIA-MS. Our spectral library supports the confident detection and quantification of proteins from the *Sus scrofa* reference proteome database with Swiss-Prot and TrEMBL sequences reduced to one per gene (Fig. 1). The library was generated from 116 DDA measurements of brain and cerebrospinal fluid (CSF) samples subjected to multiple extraction and fractionation strategies. It encompasses transitions for 89,392 peptides (78,750 proteotypic peptides) mapping to 8,321 proteins, which represents 36% of porcine protein-encoding genes. Here, we demonstrate that a hybrid DIA analysis combining the library-free and library-based approaches generates the most comprehensive and reproducible proteome-wide quantitative data. Beside DIA, this spectral library can represent a valuable resource of MS-detectable peptides for targeted quantitative analyses.

## Methods

### Tissue collection

We employed a minipig model of HD generated by the lentiviral infection of porcine embryos^12^. This TG model contains two alleles of the wild-type (WT) porcine huntingtin and one copy of a human transgene encoding the N-terminal fragment of a mutated huntingtin protein with the first 548 amino acids and 124 glutamines (mHTT). All experiments were approved by the Animal Care and Use Committee of the Institute of Animal Physiology and Genetics and the Resort Professional Commission of the Czech Academy of Sciences for Approval of Projects of Experiments on Animals (protocols no. 9/2014, 53/2015, 109/2020). The protocols align with guidelines for higher vertebrate animal studies, and particular samples used to generate the spectral library are summarised in Table 1.

**Table 1:** The overview of samples acquired by DDA-MS to generate the porcine CNS-enriched spectral library.

tissue	variable type	variable	genotype (N)	
cerebral cortex	protein extraction	RIPA on-filter	4x pool of WT & mHTT	
		SDS on-filter	4x pool of WT & mHTT	
		β-OG in-solution	4x pool of WT & mHTT	
		RIPA in-gel	4x pool of WT & mHTT	
	high-pH peptide fractionation	microsyringe	16 mHTT fractions	
		RP-HPLC	21 mHTT fraction pools	
	age	36 months	5x WT	5x mHTT
		48 months	5x WT	6x mHTT
		60 months	4x WT	4x mHTT
CSF	age	18 months	3x WT	2x mHTT
		36 months	5x WT	6x mHTT
		60 months	4x WT	4x mHTT
nucleus caudatus	age	60 months	5x WT	5x mHTT

Samples subjected to multiple extraction and fractionation strategies are shown, including their tissue, variable type, and *HTT* genotype. All peptide samples were injected once. β-OG, octyl β-D-glucopyranoside; CSF, cerebrospinal fluid; mHTT, mutated *HTT*; RIPA, radioimmunoprecipitation assay buffer; RP-HPLC, reversed-phase high-performance liquid chromatography; SDS, sodium dodecyl sulfate; WT, wild-type.

Brain tissue samples were collected as previously described^22^. Briefly, deep anaesthesia was induced with barbiturate (Thiopental Valeant, 1 g, i.v.), followed by transcardial perfusion with PBS. Brains were dissected from TG minipigs and their WT siblings, and samples from specific regions (cerebral cortex and nucleus caudatus) were snap-frozen in liquid nitrogen and stored at -80 °C.

CSF sample collection was performed as previously described^23^. Animals were fasted for 12 h before sample collection. General anaesthesia was induced with the TKX mixture (Tiletaminum 250 mg, Zolazepamum 250 mg, Ketamine 10% 3 mL, Xylazine 2% 3 mL) at a dose of 1 mL per 10 kg of body weight. CSF samples were collected by lumbar puncture. All surgeries were conducted under sterile conditions in a standard surgical room. Postoperative care included analgesics and antibiotics. Animals were housed separately during recovery from anaesthesia, then returned to the animal colony. CSF samples were centrifuged at 2,000 *g* for 10 min at 4 °C. Supernatants were transferred to new tubes, followed by further centrifugation at 2,400 *g*, and supernatants were stored at -80 °C.

### Pooled cerebral cortex samples

#### Protein extraction

The cerebral cortex samples obtained from WT (*n* = 2) and mHTT (*n* = 3) animals at the age of 24-48 months were cut into small pieces on dry ice, pooled, and mixed 1:1 (w/w) with 100 mM Tris HCl pH 7.4 for homogenisation (Miccra D1 homogeniser). The pooled sample was then divided into the aliquots of proteins that were extracted using: i) octyl β-D-glucopyranoside (β-OG) buffer (10 mM Tris HCl, 0.15 M NaCl, 1 mM EDTA and 1.25% w/v β-OG); ii) radioimmunoprecipitation assay (RIPA) buffer (150 mM NaCl, 5 mM EDTA, 0.5% v/v Igepal CA630, 1% w/v sodium deoxycholate, 1% v/v Triton X-100 and 0.1% w/v sodium dodecyl sulfate (SDS) in 50 mM Tris HCl pH 7.4); and iii) SDS buffer (2% w/v SDS in 50 mM Tris HCl pH 7.4). All chemicals were obtained from Sigma-Aldrich, except for EDTA and NaCl, which were from Carl Roth GmbH. The samples were prepared at a 1:3 (v/v) ratio of a sample to buffer, and each aliquot was further homogenised by 20 strokes in a tissue glass homogeniser (DUALL with all glass, Kimble), which was followed by sonicating on ice (10 s, 50% power; Bandelin SONOPULS ultrasonic homogeniser). The RIPA and SDS samples were heated for 5 min at 85 °C. All samples were freeze-thawed at -80 °C and centrifuged at 20,000 *g* for 20 minutes at 22 °C for the RIPA and SDS samples, or at 4 °C for the β-OG samples. Supernatants were collected, and protein concentrations were determined by the Pierce BCA protein assay kit (Thermo Fisher Scientific) according to the manufacturer’s instructions.

To control the reproducibility of a sample preparation, albumin from chicken egg white (OVAL; Sigma-Aldrich, A2512) and alcohol dehydrogenase from yeasts (ADH1; Sigma-Aldrich, A8656) were spiked into RIPA, SDS, and β-OG quadruplicates. Replicates no. 1 and 2 were at a ratio 1:60 (w/w) of the OVAL to total proteins, and at a ratio 1:400 (w/w) of the ADH1 to total proteins. Replicates no. 3 and 4 were at a ratio 1:240 (w/w) of the OVAL to total proteins, and at a ratio 1:100 (w/w) of the ADH1 to total proteins. These unfractionated protein samples were subjected to: i) in-solution digestion (β-OG samples referred to as 1 A in Fig. 1, 100 µg of proteins, *n* = 4); ii) in-gel digestion (RIPA samples referred to as 1B in Fig. 1, 45 µg of proteins, *n* = 4); and iii) on-filter digestion (RIPA and SDS samples referred to as 1 C and 1D in Fig. 1, respectively, 100 µg of proteins, *n* = 4 and 4).

#### RIPA and SDS on-filter digestion

RIPA and SDS samples were processed using our modified filter-aided sample preparation (FASP) protocol^24^. Briefly, the spiked-in RIPA and SDS samples (100 µg of proteins, *n* = 4 per condition) were diluted with a wash buffer of 8 M urea (Sigma-Aldrich) and 5 mM EDTA (Carl Roth GmbH) in 50 mM ammonium bicarbonate (AmBic; Sigma-Aldrich) to a volume of 300 μL per replicate and centrifuged at 20,000 *g* for 15 min at 20 °C. Supernatants were collected, loaded onto the Microcon 30 K centrifugal ultrafiltration units (Merck Millipore Ltd.), and centrifuged at 10,000 *g* for 30 min at 20 °C. Eluates were discarded, and the ultrafiltration units were washed three times with 200 μL of the wash buffer. Proteins were reduced with 150 µL of 10 mM tris(2-carboxyethyl) phosphine (TCEP; Sigma-Aldrich) in the wash buffer for 30 min at 32 °C and alkylated with 150 µL of 40 mM iodoacetamide (IAA; Sigma-Aldrich) in the wash buffer for 35 min at 25 °C in the dark. Eluates were discarded, and units were washed with 150 µL of the wash buffer. Units were then washed three times with 150 µL of 50 mM AmBic. Proteins were digested at 37 °C by the addition of 150 µL of 0.02% (w/v) ProteaseMAX surfactant (Promega) in 50 mM AmBic with lysyl endopeptidase (LysC; Wako Chemicals GmbH) and sequencing-grade porcine trypsin (Promega) proteases at an enzyme to substrate ratio 1:100 (w/w) for 2 h and overnight, respectively. Peptides were collected by centrifugation at 14,000 *g* for 25 min at 20 °C and eluted again with 150 µL of 50 mM AmBic. The centrifugation step was repeated, and eluates were combined. To ensure highly reproducible LC-MS/MS measurements, all RIPA and SDS on-filter (FASP) pre-cleaned samples were subjected to a general peptide cleaning step described below.

#### β-OG in-solution digestion

Using the Amicon Ultra filters (10 kDa MWCO, Merck Millipore Ltd.), the protein extraction buffer of the spiked-in β-OG samples (100 µg of proteins, *n* = 4) was exchanged for the wash buffer of 8 M urea and 5 mM EDTA in 50 mM Ambic with 0.1% (w/v) ProteaseMAX. Then, the samples were transferred to new tubes, reduced with 10 mM TCEP for 30 min at 32 °C, and alkylated with 40 mM IAA for 45 min at 25 °C in the dark. The proteins were diluted with 50 mM AmBic to a final concentration of 1 M urea, and digested at 37 °C with LysC and trypsin proteases at a ratio of an enzyme to substrate 1:100 (w/w) for 2 h and overnight, respectively. No β-OG in-solution samples were pre-cleaned by FASP, but they were subjected to the general peptide cleaning step described below.

#### RIPA in-gel digestion

The spiked-in RIPA samples (45 µg of proteins, *n* = 4) were mixed with the NuPAGE LDS sample buffer and the NuPAGE sample reducing agent (both from Thermo Fisher Scientific), heated to 70 °C for 10 min, cooled to 25 °C, and separated on the NuPAGE 3-8% Tris-Acetate gel (Thermo Fisher Scientific) for 100 min at 110 V. Each sample lane of the gel was cut into small pieces and the full molecular weight gel range was processed in one tube. The gel pieces were washed with water, shrunk by dehydration in acetonitrile, and swollen in water. The wash step was repeated, and gels were swollen in 50% (v/v) acetonitrile, which was followed by partial dehydration in a vacuum centrifuge at 45 °C (Labconco Corporation). The gel pieces were rehydrated in 10 mM TCEP for 30 min at 32 °C and in 40 mM IAA for 45 min at 25 °C in the dark. The gel pieces were washed with water-acetonitrile-water, dehydrated in the vacuum centrifuge at 45 °C, and rehydrated in 50 mM AmBic with LysC and trypsin proteases at a ratio of an enzyme to substrate 1:100 (w/w) for protein digestion at 37 °C for 2 h and overnight, respectively. No RIPA in-gel samples were pre-cleaned by FASP, but they were subjected to the general peptide cleaning step described below.

### Fractionated cerebral cortex samples

Independent β-OG (200 µg of proteins, *n* = 1) and RIPA (1,600 µg of proteins, *n* = 1) samples were processed as described above, and prior to DDA-MS, peptide mixtures were separated using a high-pH fractionation on i) microsyringe (referred to as 2 A in Fig. 1), or ii) RP-HPLC (referred to as 2B in Fig. 1).

#### High-pH RP fractionation of peptides using a microsyringe

The β-OG in-solution digest for the microsyringe high-pH RP was resuspended in 2% acetonitrile in 0.1% (v/v) trifluoroacetic acid (Sigma-Aldrich), and fractionated as described in^25^. Briefly, 30 µL of the solution with about 50 µg of peptides were injected using a 100 µL gastight microsyringe (SGE) onto the C18 microcolumn manually packed with Aeris Peptide XB, 3.6 µm, 40 mm × 250 µm (Phenomenex) at a constant pressure. A gradient of acetonitrile at high-pH was then formed by aspirating 16 µL from each of six solutions containing 20 mM ammonium formate (pH 10) and decreasing amount of acetonitrile (40%, 32%, 24%, 16%, 8%, 2%). The obtained fractions (16, each 6 µL) were collected.

#### High-pH RP-HPLC fractionation of peptides

The RIPA on-filter digests were resuspended in 20 mM ammonium formate (pH 10) and injected (400 µg of peptides, *n* = 2) into a Dionex Ultimate 300 LC system (Thermo Fisher Scientific Inc.). The peptides were separated at 60 °C on a Kinetex C18 Biphenyl column, 5 μm, 100 Å 150 × 4.6 mm (Phenomenex) at a flow rate of 0.2 mL/min by a 30-min gradient from 2% to 40% (v/v) acetonitrile in 20 mM ammonium formate (pH 10). For the first RIPA peptide sample, 32 fractions were collected (45 seconds each), 6 fractions with zero UV absorption were discarded, and the resulting 26 fractions were pooled into 13 samples by the following scheme: fraction x was pooled with fraction x + 13. For the second RIPA peptide sample, 8 fraction pools were prepared by the following scheme: fraction x was pooled with fractions x + 8, x + 16, and x + 24.

### Cerebral cortex samples of animals of different ages and *HTT* genotype

The cerebral cortex samples (~80 mg, referred to as 3 A in Fig. 1) of WT and mHTT animals sacrificed at the age of 36, 48 or 60 months were cut into small pieces on ice, mixed 1:3 (w/w) with a lysis buffer (13 mM Tris, 0.15 M NaCl, 1 mM EDTA in PBS and 1% w/v β-OG), and disrupted by 20-min vortexing at 4 °C in the presence of acid-washed glass beads. The samples were sonicated on ice for 10 min in sonication bath (Bandelin electronic GmbH & Co. KG) with ultrasonic frequency 35 kHz and centrifuged at 5,000 *g* for 5 minutes at 4 °C. Half volume of the tissue lysis buffer was added to the beads, samples were vortexed for another 10 min, sonicated for 10 min and centrifuged for 5 min at 4 °C. Supernatants containing extracted proteins were centrifuged at 20,000 *g* for 10 min at 4 °C to remove any remaining debris. Protein concentrations were determined by the Pierce BCA protein assay kit.

Using the Amicon Ultra filters (10 kDa MWCO, Merck Millipore Ltd.), the lysis buffer was exchanged for a wash buffer of 8 M urea and 5 mM EDTA in 50 mM Ambic. The samples were transferred to new tubes, supplemented with sodium deoxycholate to a final concentration of 8% (w/v), reduced with 10 mM TCEP for 30 min at 32 °C, and alkylated with 40 mM IAA for 45 min at 25 °C in the dark. The proteins were diluted with 100 mM AmBic to a final concentration of 1 M urea, and digested at 37 °C with LysC and trypsin proteases at a ratio of an enzyme to substrate 1:100 (w/w) for 2 h and overnight, respectively. No cerebral cortex samples of animals of different ages and *HTT* genotype were pre-cleaned by FASP, but they were subjected to the general peptide cleaning step described below.

### Nucleus caudatus samples of animals of different *HTT* genotype

The nucleus caudatus samples (~30 mg, referred to as 3B in Fig. 1) of WT and mHTT animals sacrificed at the age of 60 months were cut into small pieces on ice, mixed 1:10 (w/w) with the SDS buffer (2% w/v SDS in 50 mM Tris HCl, pH 7.4), and heated for 5 min at 95 °C. The samples were sonicated (10 s, 50% power; Bandelin SONOPULS ultrasonic homogeniser), and centrifuged at 20,000 *g* for 20 minutes at 22 °C. Supernatants were collected, and protein concentrations were determined by the Pierce BCA protein assay kit. The samples (100 µg of proteins) were loaded onto the Microcon 30 K centrifugal ultrafiltration units (Merck Millipore Ltd.), and centrifuged at 10,000 *g* for 15 min at 20 °C. Eluates were discarded, and the ultrafiltration units were washed three times with 200 μL of the wash buffer (8 M urea and 5 mM EDTA in 50 mM Ambic). Proteins were reduced with 150 µL of 10 mM TCEP in the wash buffer for 30 min at 32 °C and alkylated with 150 µL of 40 mM IAA in the wash buffer for 30 min at 25 °C in the dark. Eluates were discarded, and units were washed with 150 µL of the wash buffer. Units were then washed three times with 150 µL of 50 mM AmBic. Proteins were digested at 37 °C by the addition of 150 µL of 0.02% (w/v) ProteaseMAX in 50 mM AmBic with LysC and trypsin proteases at a ratio of an enzyme to substrate 1:100 (w/w) for 3 h and overnight, respectively. Peptides were collected by centrifugation at 10,000 *g* for 15 min at 20 °C, and eluted again with 150 µL of 50 mM AmBic. The centrifugation step was repeated, and eluates were combined. Nucleus caudatus samples of animals of different *HTT* genotype were pre-cleaned by FASP, and to ensure highly reproducible LC-MS/MS measurements, they were subjected to the general peptide cleaning step described below.

### Cerebrospinal fluid of animals of different ages and *HTT* genotype

Protein concentrations in CSF samples collected at the animal age of 18, 36, 48, or 60 months were determined by the Pierce 660 nm protein assay kit. The CSF protein samples (referred to as 3 C in Fig. 1) were spiked with the OVAL and the ADH1 standard proteins at a ratio of 1:100 and 1:250 (w/w) of the standard protein to sample proteins, respectively. Using the Amicon Ultra filters (3 kDa MWCO, Merck Millipore Ltd.), the samples were transferred into a wash buffer of 8 M urea and 5 mM EDTA in 50 mM AmBic, and the wash step was repeated twice. Then, the samples were transferred to new tubes, and centrifuged at 20,000 *g* for 20 minutes at 4 °C. Samples were supplemented with ProteaseMAX to a final concentration of 0.1% (w/v), reduced with 10 mM TCEP for 30 min at 32 °C, and alkylated with 40 mM IAA for 45 min at 25 °C in the dark. The proteins were diluted with 100 mM AmBic to a final concentration of 1 M urea, and digested at 37 °C with LysC and trypsin proteases at a ratio of an enzyme to substrate 1:100 (w/w) for 2 h and overnight, respectively. No cerebrospinal fluid of animals of different ages and *HTT* genotype was pre-cleaned by FASP, but these samples were subjected to the general peptide cleaning step described below.

### Peptide cleanup

All samples (including FASP samples) underwent this general peptide cleanup before injection to ensure highly reproducible LC-MS/MS measurements. The digestion of all samples was stopped by acidification with formic acid (Sigma-Aldrich) to a final concentration 2% (v/v). Samples were centrifuged at 20,000 *g* for 20 minutes at 4 °C, and supernatants were collected. Peptide mixtures from the on-filter and in-solution digestions were loaded onto C18 MacroSpin columns (The Nest Group), and peptide mixtures from the in-gel digestion were processed with ZipTip C18 (Merck Millipore Ltd.). Peptides were purified according to manufacturer´s instructions, dried using the vacuum centrifuge at 45 °C, and stored at -20 °C. Dried peptides were either prepared for the high-pH fractionation or dissolved in 2% (v/v) acetonitrile (Thermo Fisher Scientific Inc.) in 0.5% formic acid (v/v), measured at λ = 280 nm (Synergy HTX, BioTek), supplemented with iRT peptides (Biognosys), and adjusted to 0.5 or 1 µg/µL for injection.

### DDA mass spectrometry

All peptide samples (1 μg) were analysed on an Eksigent 425 nanoLC (Sciex) online coupled to a TripleTOF5600+ mass spectrometer (Sciex) as described previously^26,27^ with following modifications. The peptide mixtures were separated in a trap-elute mode with a linear gradient from 5 to 35% (v/v) acetonitrile in 0.1% (v/v) formic acid over 120 min at a flow rate of 300 nL/min, except for the pooled cerebral cortex samples and the CSF samples, where a flow rate of 200 nL/min was applied. Peptides were loaded on the C18 trap column, 5 μm, 100 Å, 20 mm × 0.1 mm (Acclaim PepMap; Thermo Fisher Scientific) at 2 μL/min for 10 min, and separated on an in-house fused-silica column, 250 mm × 75 μm (New Objective) manually packed with C18 beads (ProntoSIL, 3 μm, 120 Å; Bischoff Analysentechnik GmbH).

The mass spectrometer was operated in a positive mode as in^26,27^. Briefly, the MS1 spectra were collected in a mass to charge range from 400 to 1,250 for 300 ms in the high-sensitivity mode, with the 20 most intense ions selected for fragmentation and a dynamic exclusion of 13 s. For the high-pH RP-HPLC fractionated peptides, MS1 spectra were collected for 200 ms, and the 25 most intense ions were selected for fragmentation. Rolling collision energy was used for fragmentation. The MS2 fragment ion spectra were collected in a mass to charge range from 170 to 1,500 for 150 ms. For MS2 spectra, only fragments with a charge state from 2 to 5 were selected.

### Spectral library generation and quality control

In total, 116 DDA-MS files (Raw DDA data^28^) were processed and searched by the Pulsar algorithm implemented in Spectronaut 19.5.241126.62635 software (Biognosys) against a database derived from the porcine UniProtKB reference proteome (UP000008227; release 2024_06)^16^. The full non-redundant database with protein sequences reduced to one per gene contained 22,833 porcine proteins with the chicken OVAL (P01012), the yeast ADE1 (P00330), the human N-terminal fragment of huntingtin with the first 548 amino acids (P42858), universal contaminants (381) from^29^, and iRT peptides appended (OnePerGene DB^28^). The library (Sus scrofa library^28^) was generated from Pulsar with default BGS Factory Settings. A specific cleavage rule was created for the combination of LysC and trypsin cleavage after all lysines and after the arginine unless a proline proceeds, with two missed cleavages allowed. The carbamidomethylation of cysteine was set as a fixed modification, and protein N-terminal acetylation and methionine oxidation were set as variable modifications. A false discovery rate (FDR) cut-off 1% was applied at levels of peptide-spectrum matches, peptides, and proteins. Porcine proteins in the spectral library were annotated using a gene ontology annotation file (35497.S_scrofa, updated 22^nd^ December 2024) from UniProtKB (Gene ontology annotation^28^) (https://ftp.ebi.ac.uk/pub/databases/GO/goa/proteomes/). Parameters of compliance were tested using the DIALib-QC software tool^30^ to systematically evaluate the quality of the spectral library (complexity, characteristics, modifications, completeness, and correctness). The report is provided (DIALibQC^28^).

### DIA mass spectrometry

The RIPA and SDS on-filter peptide digests (1 μg, *n* = 4) obtained from the pooled cerebral cortex samples were analysed on the Eksigent 425 nanoLC online coupled to the TripleTOF5600+ mass spectrometer as described above for DDA-MS. Data were collected with the sequential window acquisition of all theoretical mass spectra (SWATH-MS) method, 30 overlapping variable windows calculated with the SWATH Variable window calculator (Sciex) were monitored. The MS1 and MS2 spectra for the pooled cerebral cortex were collected in mass to charge ranges corresponding to DDA-MS, and the accumulation time was set to 150 ms and 99 ms for MS1 and MS2, respectively, resulting in a cycle time of 3.2 s.

The peptide digests obtained from the 48 month old WT and mHTT animals were analysed using the same instrumentation set-up with the following modifications. For the cerebral cortices (1 μg, *n*_*WT*_ = 5, *n*_*mHTT*_ = 6), 35 overlapping variable windows were monitored, and the accumulation time was set to 99 ms and 96 ms for MS1 and MS2, respectively, resulting in a cycle time of 3.51 s. For the CSF samples (1 μg, *n*_*WT*_ = 6, *n*_*mHTT*_ = 6), 30 overlapping variable windows were monitored with the accumulation time set to 96 ms for both MS1 and MS2, resulting in a cycle time of 3.02 s. The nucleus caudatus samples obtained from the 60 month old WT and mHTT animals (0.5 μg, *n*_*WT*_ = 5, *n*_*mHTT*_ = 5) were analysed using the set-up applied to the cerebral cortices.

### DIA data processing and quantitative analysis

The DIA/SWATH-MS data (Raw DIA data^28^) were processed in Spectronaut 19.5.241126.62635 with three different analysis workflows: i) a direct DIA - library-free DIA, ii) a library DIA - library-based DIA, and iii) a hybrid DIA - direct DIA enriched for extra spectra from the search archive of DDA data used for the spectral library generation. All DIA analysis workflows were performed using default BGS Factory Settings with the following modifications. A specific cleavage rule was created for the combination of LysC and trypsin (cleavage after all lysines and after the arginine unless a proline proceeds). For the library DIA, the calibration using Biognosys’s iRT kit was selected. For the hybrid DIA, the Hybrid (DDA + DIA) Library option was selected in DIA Analysis Workflow in Experiment Settings. Data were normalised in Spectronaut using cross-run local normalisation. No filter type and automatic row selection were applied. Only RIPA and SDS samples with a qualitatively different composition caused by the different protein extraction strategy were analysed without normalisation. Differential protein abundance (Abundances^28^) analyses were performed in Spectronaut using an unpaired t-test. Statistically significant changes in protein abundance were considered with a fold-change ≥ 1.5 and Qvalue ≤ 0.05 (Pivot report^28^). Spectronaut performs quantitative analysis at the protein group level. Proteins that cannot be distinguished based on the identified peptide sequences are grouped together, and only if a protein contains at least one unique peptide, it is quantified as an individual protein group. Therefore, multiple proteins may be assigned to the same protein group, and consequently, the total number of identified proteins exceeds the number of quantified protein groups.

### Data visualisation

Protein group and peptide level quantification data for all three quantification strategies, as well as spectral library data, were exported from Spectronaut in TSV format using a custom export setting (Export schemes^28^). Further data analysis and visualisation were performed in R 4.5.1^31^. The tidyverse^32^ set of packages was used for data filtering and summarisation. The ggplot2^33^ and cowplot^34^ packages were used for graph production, the ggvd and ggVennDiagram ggplot2 extensions were used to plot Venn diagrams and the ggbeeswarm^35^ package was used to produce column scatter plots.

The porcine HTT and the N-terminal fragment of the human HTT were digested *in silico* using MS-Digest (https://prospector.ucsf.edu/prospector/cgi-bin/msform.cgi?form=msdigest), and all theoretical Lys-C/trypsin digested peptides ≥ 7 amino acids were searched in our data (HTT peptides^28^). Multiple sequence alignment was performed using Clustal Omega 1.2.4^36^ (HTT alignment^28^).

## Data Records

The raw DDA-MS data files (WIFF, WIFF.SCAN), along with the OnePerGene DB (FASTA), the spectral library (TSV, KIT), together with the gene ontology annotation (TSV) and the DIALibQC report (XLSX, PDF), the raw DIA/SWATH-MS data files (WIFF, WIFF.SCAN), the abundances (CSV), the pivot report on the robustness of the results (XLSX), the export schemes (RS), the HTT peptides (XLSX), and HTT alignment (PDF) have been deposited to the ProteomeXchange Consortium via the PRIDE partner repository^37^ with the dataset identifier PXD074603^28^.

## Technical Validation

**Fig. 2:**
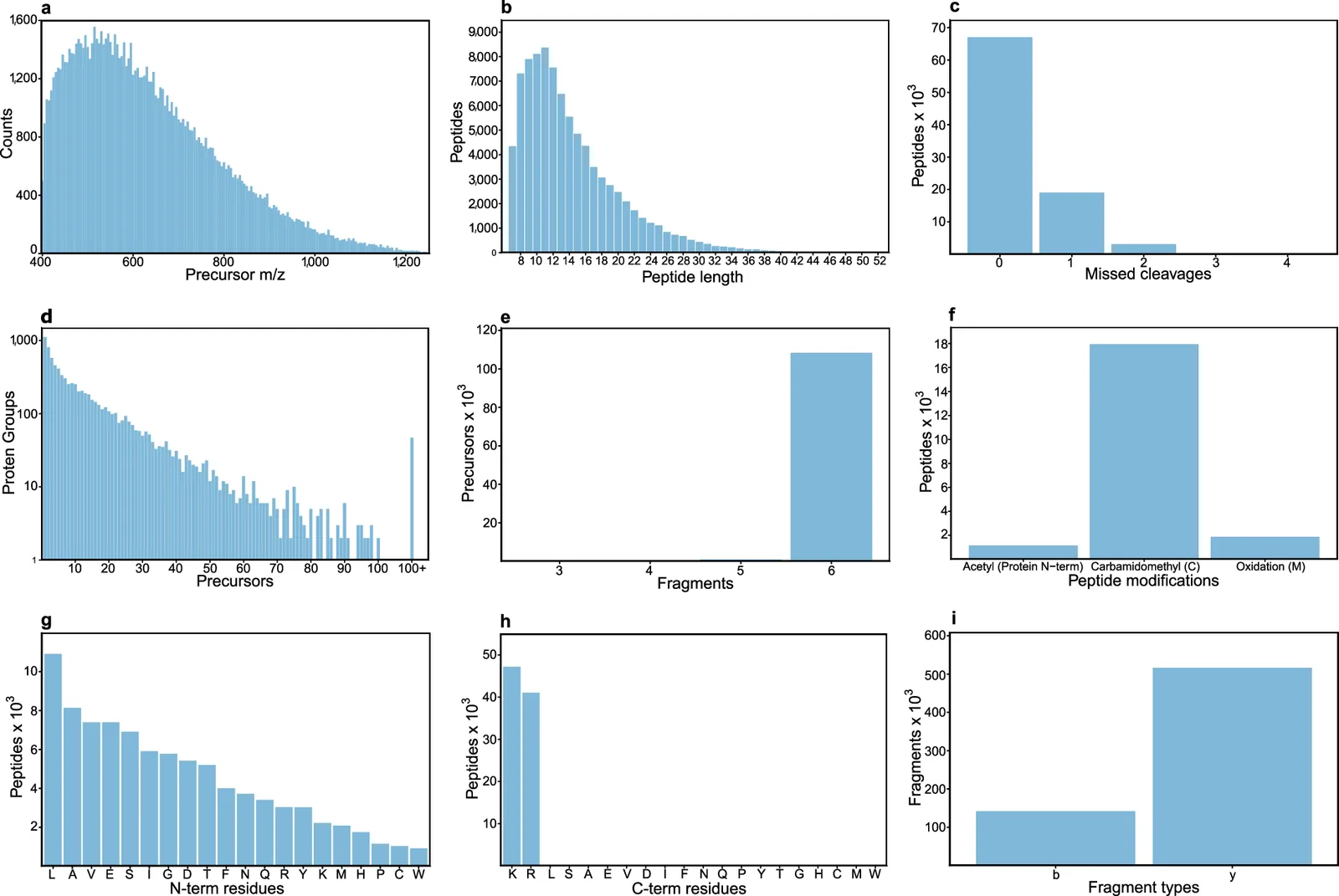
The characteristics of the porcine spectral library. (**a**) Precursor m/z distribution. (**b**) Peptide length overview. (**c**) Missed cleavages overview. (**d**) Precursors per protein group overview. (**e**) Fragments per precursor overview. (**f**) Peptide modifications overview. (**g**) N-terminal amino acid residues overview. (**h**) C-terminal amino acid residues overview. (**i**) Fragment types overview.

To generate the porcine spectral library, 116 DDA-MS injections of the brain and CSF samples were searched against the full non-redundant porcine protein database. This library encompasses assays for 89,392 peptides assigned to 8,010 protein groups, and 8,321 proteins (FDR = 0.01). Library characteristics, including the distribution of the precursor m/z (Fig. 2a), together with the overview of the peptide length (Fig. 2b) and missed cleavages (Fig. 2c), the number of precursors per protein groups (Fig. 2d) and fragments per precursors (Fig. 2e), modifications (Fig. 2f), N-terminal (Fig. 2g) and C-terminal (Fig. 2h) amino acid residues, and fragment types (Fig. 2i) demonstrate its validity^30,38,39^. At least two peptides with at least 6 fragment ions were identified for 7,204 protein groups. A library specificity is given in Table 2.

**Table 2:** The specificity of the porcine spectral library.

organisms	protein groups	peptides	note
*Sus scrofa*	7,941	88,754	
*Bos taurus*	32	208	contaminants
*Homo sapiens*	20	245	contaminants, human HTT fragment
*Bos taurus*; *Sus scrofa*	6	63	contaminants with common peptide with *Sus scrofa* protein
*Homo sapiens*; *Sus scrofa*	2	2	contaminants with common peptide with *Sus scrofa* protein
*Homo sapiens*; *Mus musculus*	1	3	contaminants, keratin
*Mus musculus*	1	3	contaminants, keratin
*Achromobacter lyticus*	1	18	contaminants, LysC
*Escherichia coli* (strain K12)	1	2	contaminants, Beta-galactosidase
*Hevea brasiliensis*	1	1	contaminants, Small rubber particle protein
*Oryctolagus cuniculus*	1	29	contaminants, Fructose-bisphosphate aldolase A
*Gallus gallus*	1	29	spike-in protein
*Saccharomyces cerevisiae* (strain ATCC 204508 / S288c)	1	22	spike-in protein
iRT peptides	1	11	

To apply the library to porcine brain deep proteome research, we selected three relevant groups from the samples used to generate our library and measured them in DIA/SWATH-MS mode. These groups included the comparison of i) the RIPA (*n* = 4) and SDS (*n* = 4) on-filter digests from the pooled sample with expected broad changes in protein identifications and quantification due to the different protein extraction strategy, but low measurement variability within measured replicates (technical replicates); ii) the cerebral cortex of WT (*n* = 5) and mHTT (*n* = 6) animals (48 months old); and iii) the nucleus caudatus of WT (*n* = 5) and mHTT (*n* = 5) animals (60 months old) with expected moderate changes in protein quantities between conditions and significant biological variability. An additional group has been represented by CSF samples from WT (*n* = 6) and mHTT (*n* = 6) animals (48 months old) that were not used to generate the library. In this fourth independent group of CSF samples with typical dynamic protein composition and different sample complexity (compared to the cortex and nucleus caudatus), we expected the highest variability.

**Fig. 3:**
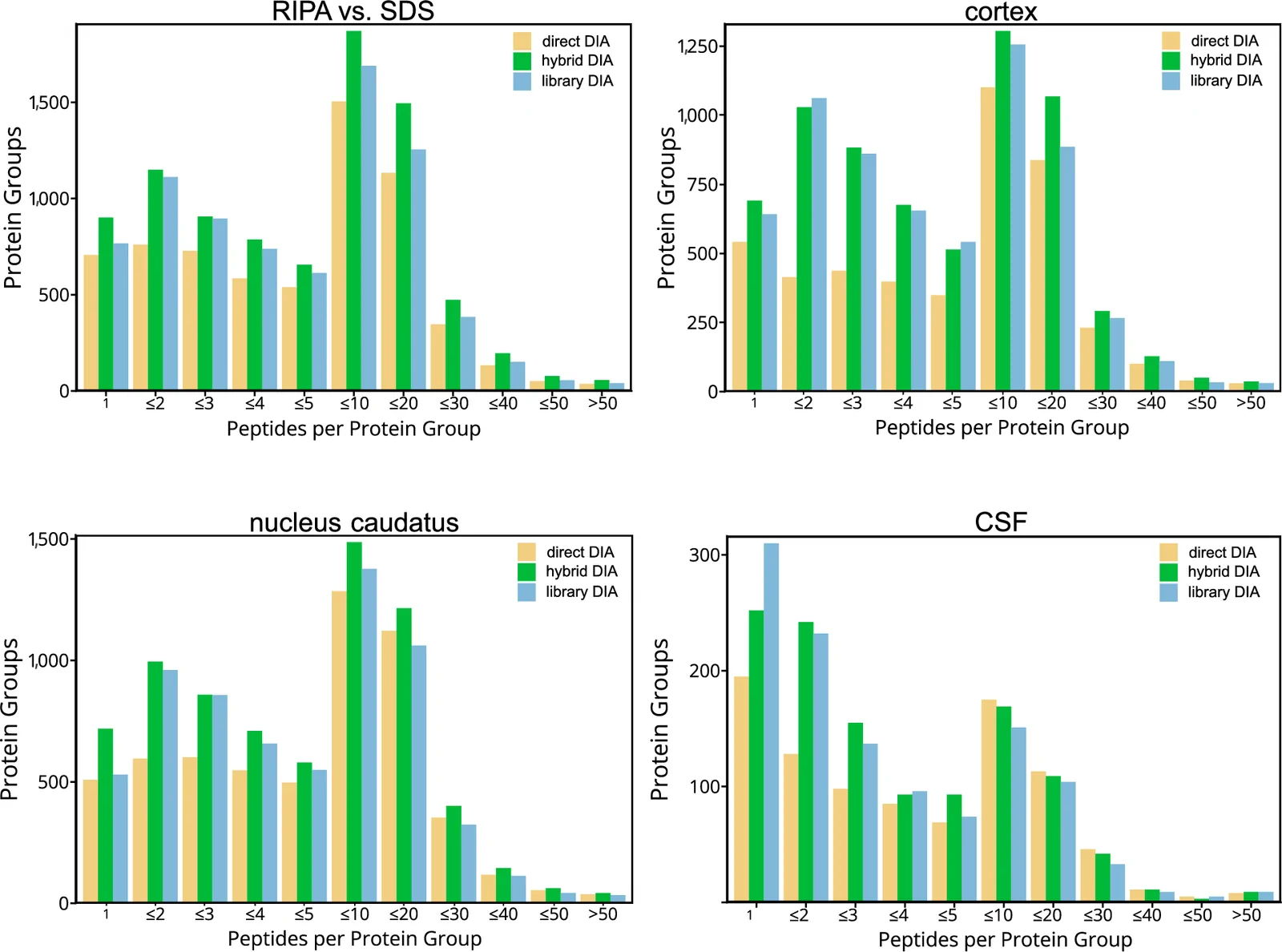
The porcine spectral library impact on the peptide enrichment.

The DIA/SWATH-MS data were processed with i) a library-free approach (direct DIA); ii) a library-based approach (library DIA); and iii) the combination of direct DIA and library DIA (hybrid DIA). Our data show that the employment of spectral library resulted in an enrichment of quantified peptides. Mainly proteins with less than 5 quantifiable peptides were affected (Fig. 3). Compared to direct DIA, hybrid DIA was enriched with quantified peptides in all groups (Table 3), including the RIPA and SDS replicates (40% increase), the cerebral cortex bioreplicates (59% increase), the nucleus caudatus bioreplicates (36% increase), and the CSF bioreplicates (19% increase).

**Table 3:** The improved protein quantification in the hybrid DIA.

conditions			protein groups		quantified peptides		differential abundance candidates		conditions intravariability (coefficient of variation)	
			direct DIA	hybrid DIA	direct DIA	hybrid DIA	direct DIA	hybrid DIA	direct DIA	hybrid DIA
RIPA vs. SDS			4,959	5,542	47,704	66,774	45.3%	48.1%	11.3% vs. 9.5%	11.0% vs. 9.8%
WT vs. mHTT	brain	cortex	4,149	5,238	39,658	63,073	2.3%	2.1%	18.9% vs. 16.4%	17.4% vs. 15.4%
		nucleus caudatus	4,973	5,660	51,877	70,620	0	0	26.2% vs. 23.0%	24.3% vs. 21.3%
	CSF		862	959	6,845	8,159	1.0%	0.6%	31.2% vs. 33.5%	29.5% vs. 30.8%

The direct DIA and hybrid DIA results summary was extracted from Spectronaut sparse profiles with peptides identified in at least one sample (Pivot report^28^). Differential abundance candidates are protein groups with a fold-change ≥1.5 and Qvalue ≤ 0.05.

As expected, the most pronounced protein changes were reported for the RIPA-SDS comparison (48.1% differentially abundant proteins, Table 3). Moderate protein changes were typical for the effect of different *HTT* genotype in the cerebral cortex bioreplicates (2,1% differentially abundant proteins), only sporadic protein changes were detected in the CSF samples (0.6% differentially abundant proteins), and no significant changes were found in the nucleus caudatus bioreplicates (Table 3). The lowest median coefficient of variation (CV) was reported for the SDS and RIPA technical replicates, which was followed by the cerebral cortex, nucleus caudatus, and CSF WT and mHTT bioreplicates (Table 3). Compared to direct DIA, WT and mHTT bioreplicates intravariability slightly decreased in hybrid DIA (~2% lower median CV, Table 3) with increased proteome coverage.

**Fig. 4:**
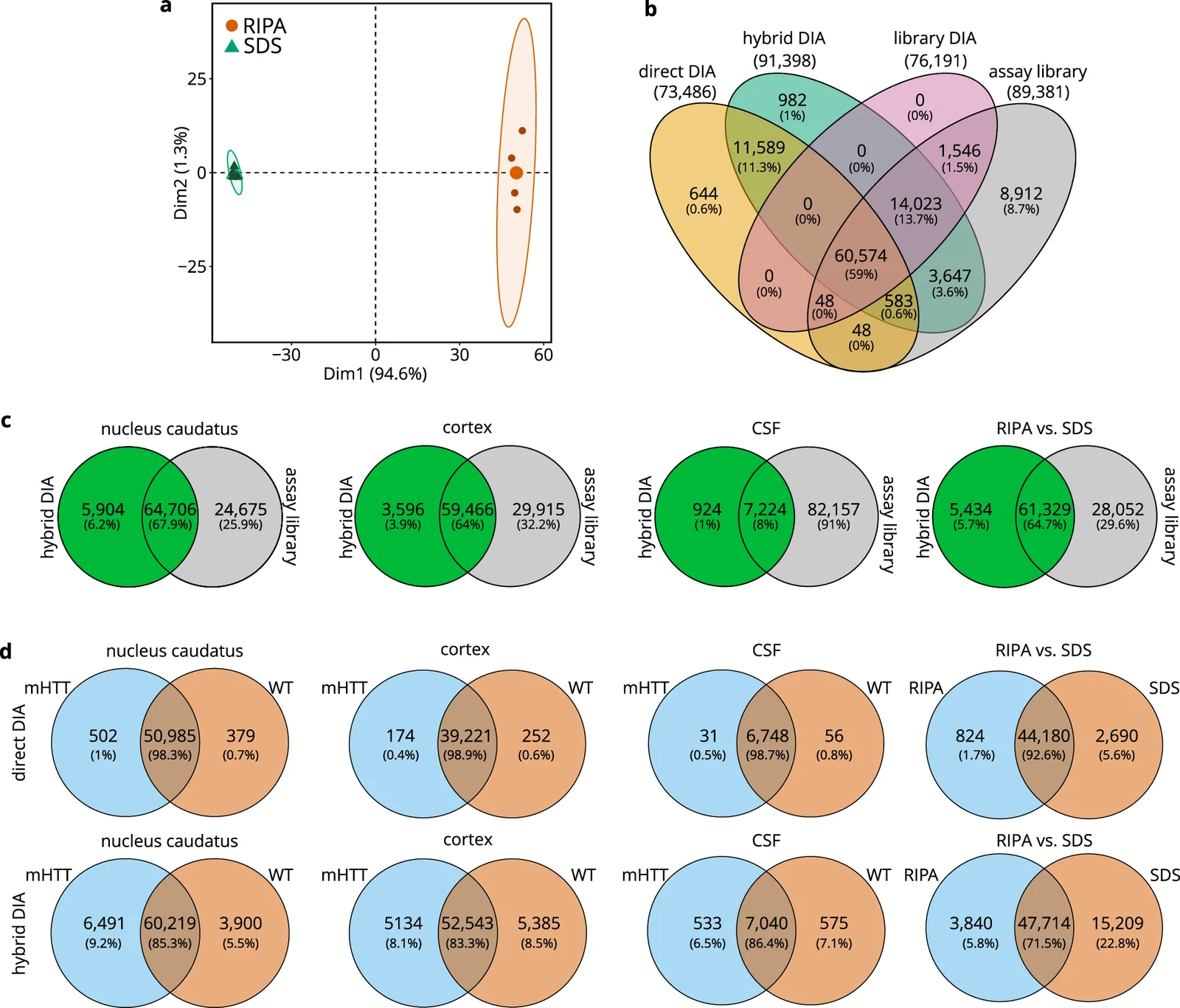
The application of the porcine spectral library. (**a**) Principal component analysis of RIPA-SDS samples quantified by the hybrid DIA. (**b**) Unique peptides overlaps for different DIA data analysis strategies applied to all conditions quantified in this study. (**c**) The coverage of sample-specific peptides quantified by the hybrid DIA. (**d**) Unique peptides overlaps for different sample conditions.

Only the RIPA and SDS samples were clearly resolved by principal component analysis (Fig. 4a). Most peptides (59.1%) were shared between the direct DIA, library DIA, hybrid DIA and the spectral assay library, the vast majority (87.1%) were found in our library, and a considerable overlap (11.3%) was demonstrated exclusively for the direct DIA and hybrid DIA (Fig. 4b). The complex samples (nucleus caudatus, cortex, and RIPA-SDS) appeared essential for the spectral library (Fig. 4c). The CSF samples that were not used for the generation of the library (48 months old WT and mHTT bioreplicates) exposed only 7.9% of peptides from the spectral library (Fig. 4c). The overlap for WT and mHTT samples was more than 98% and 83% of peptides for the direct DIA and hybrid DIA, respectively (Fig. 4d). For the RIPA-SDS samples with the different extraction strategy and the 48.1% differentially abundant proteins, this overlap was 92.6% and 71.5% of peptides for the direct DIA and hybrid DIA, respectively (Fig. 4d).

**Fig. 5:**
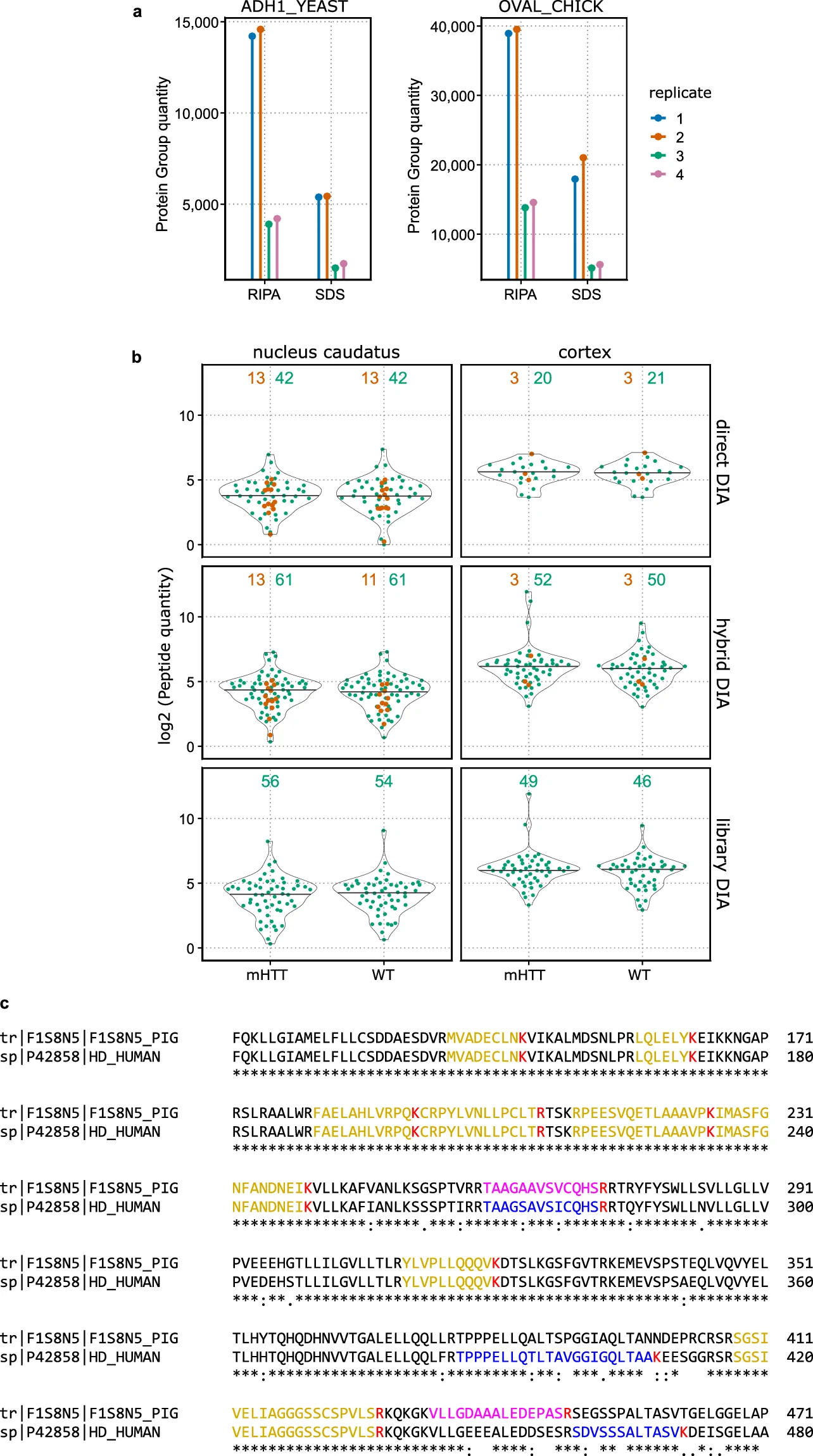
The DIA/SWATH-MS data extraction of targeted proteins. (**a**) The yeast ADH1 and the chicken OVAL standard proteins in the RIPA-SDS technical replicates (duplicates dilution at the ratio of 4:1 and 1:4) quantified using hybrid DIA. (**b**) Peptides of the porcine HTT protein in the nucleus caudatus and cortex samples analysed by different DIA workflows. Peptides present in the spectral library are displayed in green, peptides detected only in the direct or hybrid DIA approach are in orange. (**c**) Zoomed protein sequence alignment of the porcine and human HTT (HTT alignment^28^). Coloured labels indicate the peptides quantified using the hybrid DIA in the RIPA-SDS comparison. Blue peptides are unique for the human HTT, pink peptides are unique for the porcine HTT, yellow peptides are identical in both the porcine HTT and the human HTT. All HTT peptides from the library are given along with the conditions that enabled their quantification (HTT peptides^28^).

To validate our library at the specific protein level, we extracted the chicken OVAL and the yeast ADE1 proteins, the human N-terminal fragment of mHTT, and the porcine HTT proteins from the DIA/SWATH-MS data, and examined their abundances. The chicken OVAL and the yeast ADE1 were spiked into the RIPA and SDS replicates and their level was evidenced to reflect the dilution of these standards (Fig. 5a). These two non-mammalian proteins were added into the RIPA and SDS sample duplicates within the observed conditions at the ratio of 4:1 and 1:4, and the quantification results confirmed such trends in abundance differences in the relevant samples (Fig. 5a). The CSF conditions with the equal dilution of the chicken OVAL and the yeast ADE1 in all bioreplicates revealed that the 47%, 47%, and 52% protein coverage of the chicken OVAL, and 55%, 62%, and 62% protein coverage of the yeast ADE1 were achieved using the direct DIA, library DIA, and hybrid DIA approaches, respectively.

Next, we searched for the porcine WT HTT and the human mHTT proteins that have 91% homology within the N-terminal fragment of interest (the first 548 amino acids). In our library, we found 46.9% of all 177 theoretical HTT tryptic peptides, out of which, 81 peptides were assigned to the porcine HTT, and two peptides were assigned to the human HTT (HTT peptides^28^). We confirmed the presence of the porcine WT HTT in cerebral cortex and nucleus caudatus bioreplicates, and its increased protein coverage in the hybrid DIA (Fig. 5b). Using the hybrid DIA, the human mHTT was detected only in the RIPA and SDS samples with eight quantified peptides shared between the human mHTT and porcine HTT (Fig. 5c). Importantly, the RIPA extraction and hybrid DIA enabled us to quantify other three peptides unique for the human mHTT protein, and two peptides unique for the porcine HTT protein (Fig. 5c). The comparison of DIA approaches in RIPA-SDS conditions revealed that the direct DIA, library DIA, and hybrid DIA achieved the 18%, 22%, and 28% protein coverage of the human mHTT (N-terminal part), respectively. The 20%, 26%, and 31% protein coverage of the porcine WT HTT (full sequence) was achieved in the identical comparison.

In conclusion, we present a novel spectral assay library derived from porcine brain samples. With the currently deepest coverage of the porcine CNS proteome, our library strengthens the quantitative potential of untargeted proteomic studies and provides a comprehensive resource for the analysis of porcine CNS-derived samples. We also report on protein extraction and peptide fractionation strategies and their potential for the development of quantitative mass spectrometry assays.

## Usage Notes

The validation of our porcine CNS-derived spectral library highlights its benefits for porcine proteomic research, particularly for brain tissues. Samples with lower protein complexity, such as CSF, should be analysed with caution, as an effort to assign peptides using comprehensive spectral libraries carries the risk of generating false positive matches. For these samples, a direct DIA or sample-specific library appears more suitable.

The presented library may be applied in DIA data analyses using analogous software, including DIA-NN (version 2.0 tested), Skyline (version Skyline-daily 25.1.1.271 tested), and Spectronaut. Comprehensive spectral libraries are instrumental for the targeted MS methods, such as selected reaction monitoring (SRM) and parallel reaction monitoring (PRM), as they allow the selection of peptides with verified detectability by LC-MS/MS^40^. Our library may be employed to develop such targeted MS methods.

The spectral library created with OnePerGene DB is convenient for general use. For targeted MS studies focused on particular protein species (e.g., tissue-specific protein variants), a spectral library generated from the original porcine UniProtKB reference proteome database can be beneficial. It is important to note that, compared to the OnePerGene DB, the full UniProtKB reference proteome database suffers from a limited number of proteotypic peptides, and a lack of protein annotation.

To maximise the proteome coverage and depth of the porcine spectral library, multiple protein extraction methods and peptide fractionation strategies were applied to pooled WT and mHTT cortex samples. However, such pooling may increase the complexity of DDA spectra, bias the library composition towards highly abundant peptides shared across conditions, and reduce the representation of condition-specific peptides relevant to this disease model. Therefore, individual biological replicates (non-pooled samples from WT and mHTT-expressing animals) were included for all analysed tissues, including cortex, nucleus caudatus, and CSF. To facilitate studies focused on WT- or mHTT-specific peptide signatures, all raw data are provided, enabling reanalysis of selected samples and generation of condition- or tissue-specific porcine spectral libraries. Such tailored libraries may be particularly valuable for low-complexity samples such as CSF.

## Data Availability

The spectral library, including all related files, is available at the ProteomeXchange Consortium via the PRIDE partner repository with the dataset identifier PXD074603^28^.

## Code availability

No custom code has been used to create the spectral library. The R code (R version 4.5.1) used to generate graphs presented in Figs. 2–5 is available upon request.
